# 148. Implementation of Restriction Criteria within an Electronic Medical Record and Its Impact on Carbapenem Prescribing

**DOI:** 10.1093/ofid/ofab466.350

**Published:** 2021-12-04

**Authors:** Mandana Naderi, Kimberly Welker, Gary Chan, David Nix

**Affiliations:** 1 University of Arizona College of Pharmacy, Phoenix, Arizona; 2 Mercy Gilbert Medical Center, Gilbert, Arizona; 3 Mercy San Juan Medical Center, Carmichael, California

## Abstract

**Background:**

Carbapenem restriction criteria (CRC) were developed by our health system to conserve the prescribing of these broad-spectrum agents. The purpose of this study was to compare pre and post EMR implementation of adherence to the system-approved CRC and if there was an association with decreased utilization of carbapenems.

**Methods:**

A retrospective cohort review from January 2018 to June 2020 was performed via the Cerner EMR at 3 community hospitals in Arizona (AZ) and California (CA) to determine if CRC was appropriate at time of carbapenem initiation. Admitted patients > 18 years prescribed meropenem or ertapenem and received at least one dose were included. Health System approved CRC are shown in Table 2.

**Results:**

A total of 160 patients were analyzed, including 60 pre-EMR CRC intervention and 100 post intervention. Forty-five patients (28%) had a documented history of ESBL infection as shown in Table 1. Figure 1 shows carbapenem utilization over the study period. An interrupted time series analysis was performed for both AZ and CA. After correcting for pre-intervention trends, AZ days of therapy (DOT) decreased by 6.2 DOT per 1000 patient days within 1 month post intervention (23%, p< 0.0001); the model predicted a further drop of 0.6 DOT per 1000 patient days per month over the 6 months post intervention. The CA DOT decreased by 1.2 DOT per 1000 patient days 1 month post intervention (17%, p= 0.28), with a predicted further drop of 0.28 DOT per 1000 patient days per month over the 6-month period post intervention. Post implementation retrospective review as described in Table 2 aligned with EMR restriction criteria selection for 68% of patients; interfacility differences occurred with 96% of CA reviews supported by criteria and 59% of AZ reviews supported by criteria (p= 0.0025).

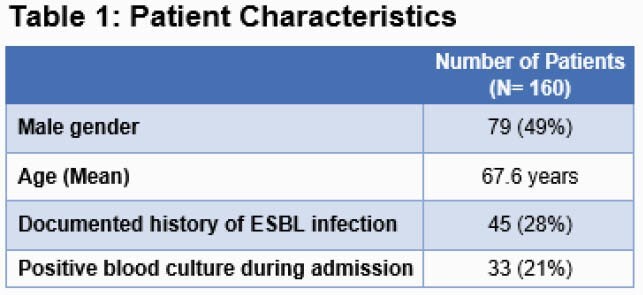

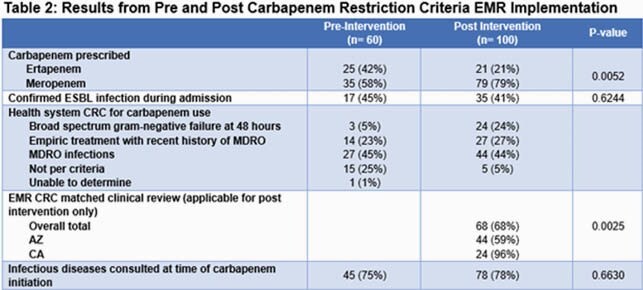

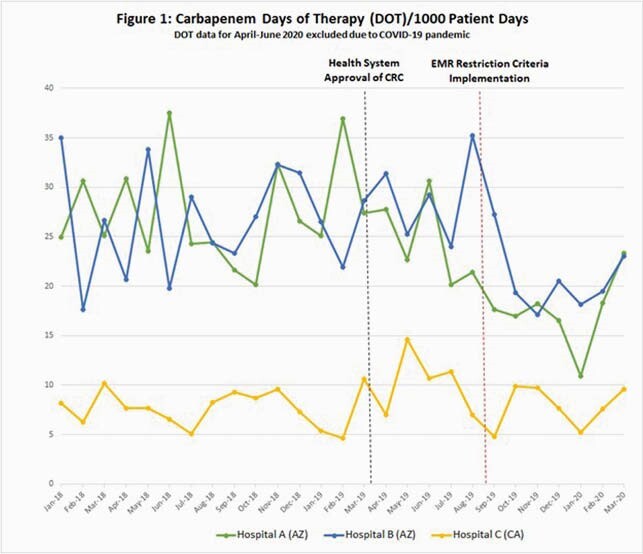

**Conclusion:**

This analysis supports that implementation of an EMR tool is an effective intervention to decrease unnecessary carbapenem use at the time of prescribing. The ESBL rate was similar pre and post intervention which may indicate that decreases in DOT were not due to a difference in MDRO rate. This study also highlights the different baseline antibiotic prescribing practices that may exist between facilities.

**Disclosures:**

**All Authors**: No reported disclosures

